# Large Gatherings? No, Thank You. Devaluation of Crowded Social Scenes During the COVID-19 Pandemic

**DOI:** 10.3389/fpsyg.2021.689162

**Published:** 2021-05-31

**Authors:** Claudia Massaccesi, Emilio Chiappini, Riccardo Paracampo, Sebastian Korb

**Affiliations:** ^1^Department of Clinical and Health Psychology, University of Vienna, Vienna, Austria; ^2^Netherlands Institute for Neuroscience, Royal Netherlands Academy of Art and Sciences (KNAW), Amsterdam, Netherlands; ^3^Department of Psychology, University of Essex, Colchester, United Kingdom; ^4^Department of Cognition, Emotion, and Methods in Psychology, University of Vienna, Vienna, Austria

**Keywords:** COVID-19, social distancing, social gatherings, valence, arousal, perceived physical distance

## Abstract

In most European countries, the first wave of the COVID-19 pandemic (spring 2020) led to the imposition of physical distancing rules, resulting in a drastic and sudden reduction of real-life social interactions. Even people not directly affected by the virus itself were impacted in their physical and/or mental health, as well as in their financial security, by governmental lockdown measures. We investigated whether the combination of these events had changed people's appraisal of social scenes by testing 241 participants recruited mainly in Italy, Austria, and Germany in an online, preregistered study conducted about 50 days after the beginning of the COVID-19 outbreak in Europe. Images depicting individuals alone, in small groups (up to four people), and in large groups (more than seven people) were rated in terms of valence, arousal, and perceived physical distance. Pre-pandemic normative ratings were obtained from a validated database (OASIS). Several self-report measures were also taken, and condensed into four factors through factor analysis. All images were rated as more arousing compared to the pre-pandemic period, and the greater the decrease in real-life physical interactions reported by participants, the higher the ratings of arousal. As expected, only images depicting large gatherings of people were rated less positively during, compared to before, the pandemic. These ratings of valence were, however, moderated by a factor that included participants' number of days in isolation, relationship closeness, and perceived COVID-19 threat. Higher scores on this factor were associated with more positive ratings of images of individuals alone and in small groups, suggesting an increased appreciation of safer social situations, such as intimate and small-group contacts. The same factor was inversely related to the perceived physical distance between individuals in images of small and large groups, suggesting an impact of lockdown measures and contagion-related worries on the representation of interpersonal space. These findings point to rapid and compelling psychological and social consequences of the lockdown measures imposed during the COVID-19 pandemic on the perception of social groups. Further studies should assess the long-term impact of such events as typical everyday life is restored.

## Introduction

The year 2020 has been marked, in most regions of the world, by the COVID-19 pandemic and its accompanying devastating effects on the economy and on individuals' physical and mental health. To protect the economy and prevent the collapse of health systems, most governments have adopted radical and unprecedented measures (see Supplementary Material for a list and a timeline of governmental measures introduced in Austria, Germany, and Italy). These included drastically reducing citizens' real-life social interactions, by limiting their freedom of movement and social exchange (social physical distancing). In the most extreme cases, people without family spent several months alone, without any meaningful physical social interactions.

This prevalence of prolonged isolation is worrisome, as humans possess “a pervasive drive to form and maintain at least a minimum quantity of lasting, positive, and impactful interpersonal relationships” (Baumeister and Leary, 1995, p. 497). A lack of social connections, and the resulting social isolation, has negative consequences on mental and physical health (Cacioppo et al., 2011, 2015), as the quality and quantity of social ties represent a major predictor for susceptibility to disease and mortality (Snyder-Mackler et al., 2020). According to the social buffering hypothesis, social support plays a crucial role in mitigating the negative consequences of adverse experiences (Cohen and Wills, 1985). Moreover, not only close others, but also weak ties, i.e., interactions with people on the periphery of the social network, seem to contribute to social and emotional well-being (Sandstrom and Dunn, 2014). Therefore, social networks may represent a crucial resource for resilience and survival in times of crisis, such as the current pandemic situation. Accordingly, greater social connectedness has been found to act as a buffer against perceived stress during the lockdown period (Nitschke et al., 2021) and to influence trust and adherence to governments' safety rules (Lamarche, 2020).

However, while gregariousness, i.e., the tendency to seek the company of others, has a beneficial impact on individuals' well-being, it also carries infection-specific risks (Schaller, 2011). During typical times, the social benefits outweigh the costs of pathogens transmission. Nonetheless, in times of high vulnerability, individuals may become less prone to sociality, to protect themselves from possible sources of infection (Schaller, 2011). Throughout the 2020–2021 pandemic, and especially during its initial phase, the importance of physically distancing from others has been emphasized by official government announcements and reminded relentlessly by major media outlets. In addition, many media reported daily (sometimes hourly) the continuously rising numbers of coronavirus-related infections and deaths, which made the topic of COVID-19 even more prevalent—shaping the social representation of the pandemic situation (Papapicco, 2020)—and likely contributed to a general feeling of fear and anxiety that influenced individuals' attitude toward health-related behaviors (Bendau et al., 2021). As a result, even brief social encounters previously perceived as trivial and insignificant—such as passing next to an unknown person in the aisle of a supermarket—took on a connotation of immediate, life-threatening danger.

Given the drastic changes in social interactions and proximity behaviors that occurred during the initial phase of the pandemic, we wondered whether people's appraisal of social scenes had changed as compared to the pre-pandemic period. Specifically, we hypothesized a change in the perceived connotation of scenes depicting large gatherings of people. These scenes, which before the pandemic outbreak were commonly associated with positive emotions, would now have become the cause of negative thoughts and a signal of a potentially dangerous situation. Moreover, we aimed to understand if such potential changes in the perception of large social gatherings could be moderated by the degree to which one had become personally affected by COVID-19, in terms of health, psychological, and financial impact, or increased loneliness.

To investigate these phenomena, we asked participants in several European countries to provide us with a range of information relating to their current and past living condition and their experience with the pandemic, and to rate the valence, arousal, and perceived physical distance of images depicting either a single person, small groups of people, or large social gatherings. The same images had been rated before the COVID-19 outbreak by another group of participants with comparable age and gender distribution (Kurdi et al., 2017), allowing a comparison of the appraisal of social gatherings before and after the pandemic outbreak.

## Materials and Methods

### Subjects

We set the goal to test at least 110 participants in a 2-week period, corresponding to the number of participants who rated each image in the pre COVID-19 study by Kurdi et al. (2017). In total, 383 participants took part in the study, of which, however, only 241 completed it in all its parts. Three participants were excluded, as they failed to rate more than one-third of the items in one or more rating scales. Thus, the final sample included 238 participants (see Table 1 for demographics). All participants were over 18 years old and provided consent to the use of the collected anonymous data. They were recruited via advertisements posted on social media (e.g., Facebook) or via direct contact (e.g., email). Participation was voluntary, and participants were not given any incentive for their participation. The study was approved by the Ethics Review Board of the University of Amsterdam (2020-EXT-12259).

**Table 1:** Demographics of the samples that rated valence and arousal of the stimulus images before (Kurdi et al., 2017; left column), and during the COVID-19 pandemic (current study; right column).

	**Before COVID-19**	**During COVID-19**
*N*	818	238
Nationality	Unknown	Italian (56%), German (14%), Austrian (9%), Dutch (1%), Other (20%)
Country of residence	USA (100%)	Italy (48%), Austria (22%), Germany (10%), UK (2%), Other (18%)
Gender	Male 49%, Female 51%	Male 37%, Female 62%, Other 1%
Age	M = 36.6; SD = 11.9; range 18–74	M = 35.4; SD = 13.6; range 20–82

### Stimuli

A set of 60 images of social scenes (see Supplementary Material for a complete list) was selected from the database Open Affective Standardized Image Set (OASIS; Kurdi et al., 2017). The essential criterion for the selection was the presence of people, although images with sexual (e.g., explicit nudes, sexual activity), medical (e.g., surgery, injections), or grisly (e.g., wounds, violent scenes, cadavers) content were excluded. Depending on the number of people depicted, images were split into three categories: Alone (one person only), Small group (two to four people), Large group (more than seven people). Images were matched across categories for valence (the degree of positive or negative affective response that the image evokes) and arousal (the intensity of the affective response that the image evokes) according to the normative values of the OASIS database (see Table 2).

**Table 2:** Normative values of mean (SD) valence and arousal for the images included, as reported in the OASIS database.

	**Alone**	**Small group**	**Large group**
Valence	5.1 (0.48)	5.27 (0.47)	5.11 (0.45)
Arousal	3.88 (0.49)	3.74 (0.54)	3.67 (0.58)

### Procedure

Data were acquired anonymously online (with the platform www.soscisurvey.de; Leiner, 2019), from April 30 to May 15. At the beginning of the experiment, participants could choose to view all materials (informed consent, instructions, task, and questionnaires) in either English, German, or Italian. After reading the instructions and providing consent, participants provided demographic data. Then they responded to a series of questions (see Supplementary Material for details) about (1) whether they were currently self-isolating, or had done so in the past, and for how long; (2) the number of people they (had) isolated with; (3) how their physical and virtual contacts with friends and relatives had changed compared to the pre-pandemic period; (4) whether they felt closer to friends and relatives compared to the pre-pandemic period; and (5) whether they or somebody among their family or friends had been diagnosed with COVID-19, and if so what the health consequences had been. Participants also filled out the “Perceived Coronavirus Threat” and the “Coronavirus Impacts” questionnaires (Conway et al., 2020).

**Figure 1:**
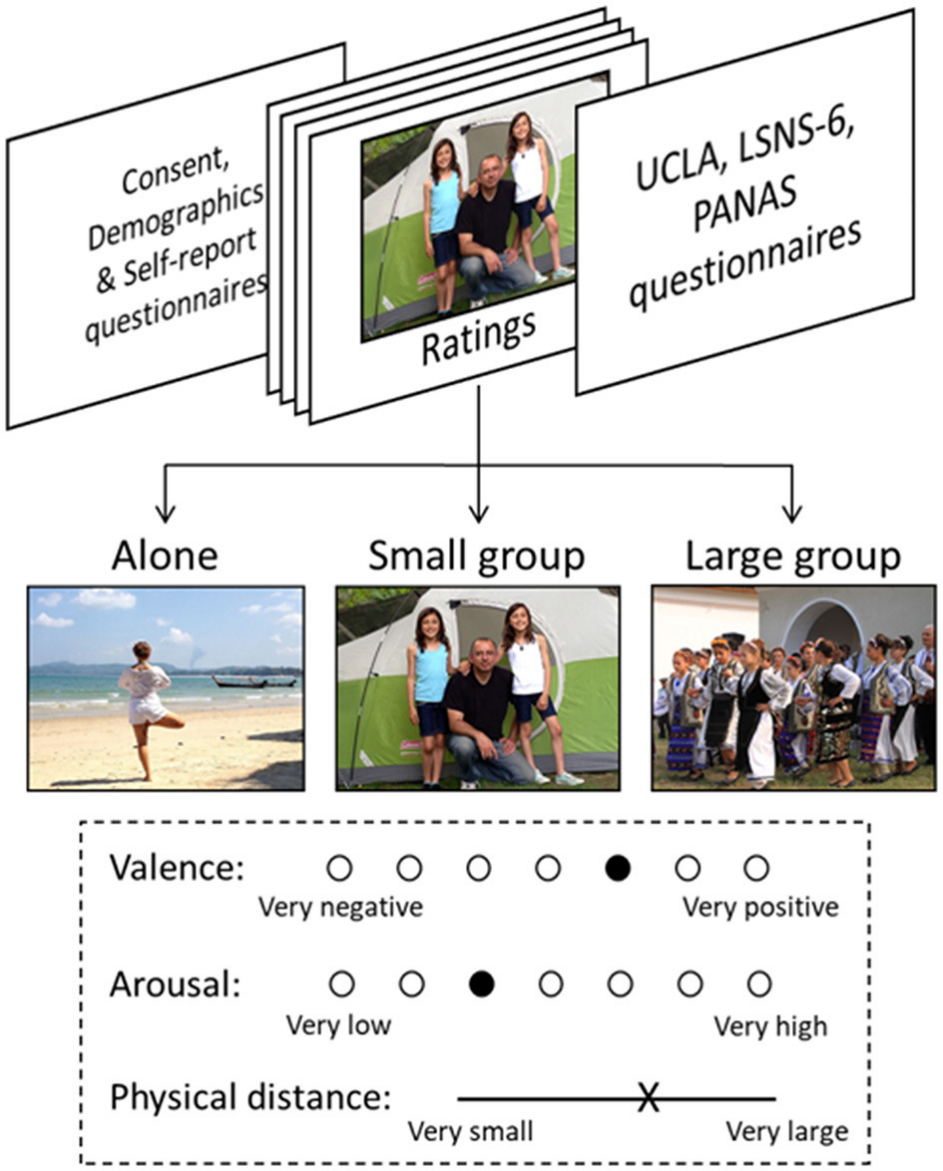
Overview of the study and examples of the three categories of images: Alone, Small group, and Large group. Ratings of perceived physical distance between people were collected only for images depicting more than one individual. Images are from the OASIS database (Kurdi et al., 2017).

In the main task (see Figure 1), participants saw the images one at a time, in random order, and rated their intrinsic valence and arousal using a seven-point Likert scale, as well as the perceived physical distance between the people shown on a visual analog scale (100 continuous points, this rating was not collected for images of the “alone” category). Every image was seen and rated once per participant.

After completing the picture rating task, participants filled out the UCLA Loneliness Scale (Russell, 1996), the abbreviated Lubben Social Network Scale (LSNS-6; Lubben et al., 2006), and the Positive Affect and Negative Affect Scales (PANAS; Watson et al., 1988). The total duration of the study was ~25 min.

### Statistical Analyses

The analysis plan was preregistered on the public data repository Open Science Framework (https://osf.io/mqau2/). Data collection had already begun at the time of preregistration to readily capture the ongoing phenomenon, yet data processing and analysis had not.

To investigate whether the COVID-19 pandemic induced changes in the evaluation of social images, we had originally planned to conduct a Linear Mixed Model (LMM) for each dependent variable (ratings of valence, arousal), with Group Numerosity (alone, small group, large group), and Time (pre COVID-19 outbreak: data from Kurdi et al., 2017, post COVID-19 outbreak: current study) as fixed effects. This analysis plan was subsequently revised, to account for minor differences between our experimental design and the one used by Kurdi et al. (2017); where each subject rated only a subset of the images, see further explanation in the Supplementary Materials. Thus, we normalized (*z*-scores transformation) the ratings of valence and arousal collected in our study using the mean and standard deviation of the ratings of the same images collected in 2017. We then fitted to these z-scores of valence and arousal two separate LMMs, using the function *lmer* of the package *lme4*, with as fixed effect the within-subjects factor Group Numerosity (alone, small group, and large group), and as random effects by-subject intercepts and Group Numerosity slopes (the summary tables of all models are provided in the Supplementary Materials).

We conducted an exploratory factor analysis using principal component analysis with varimax rotation on the following measures: the UCLA Loneliness Scale (Russell, 1996), the abbreviated LSNS-6 (Lubben et al., 2006), changes in physical and virtual interactions, changes in feelings of closeness, the “Perceived Coronavirus Threat” and the “Coronavirus Impacts” questionnaires (Conway et al., 2020), and the number of days in isolation. Participants who indicated not to have been isolating (neither in the present nor in the past) were assigned a value of zero isolation days. We excluded participants (*N* = 5) who reported to have been isolating in the past, as we did not assess how long before they had been in isolation. We first used a parallel analysis and scree plot to determine the number of factors for the exploratory factor analysis, which both revealed the presence of three factors. However, since factor-4 presented an eigenvalue of one and the variable constituting it (COVID-19 impact) had a low loading (0.39) when using three factors only, we opted for a four factors structure, accounting for 69% of the total variance (as opposed to 57% of the three factors structure). The identified factors were subsequently added, as fixed main and interaction effects, to the previously described LMMs. The same analyses were carried out on the collected ratings of physical distance.

To control for individual differences, we also included in all LMMs the covariates age, nationality, country of residence, and participants' personal experience with COVID-19 (see Table 3). In all cases, continuous predictors were mean-centered and scaled. Where relevant, *post-hoc* analyses were adjusted for multiple comparisons using Tuckey correction.

**Table 3:** Participants' personal experience with COVID-19.

**Personal experience with COVID-19**			
Have you or anybody in your circle of		Consequences:	
acquaintances been tested positive			
for COVID-19?^*^			
1. No	61%	—	
2. Yes, somebody in my circle of acquaintances	38%	a. No serious consequences	49%
		b. Hospitalization	31%
		c. Intensive care	6%
		d. Death	12%
		e. Prefer not to answer	2%
3. Yes, myself	0%	—	—
4. Yes, myself and somebody in my circle of acquaintances	1%	a. No serious consequences	100%

* *Only one of the responses 1–4 could be chosen*.

The dataset and the analysis script in R are available at https://osf.io/mqau2/.

## Results

### Subjective Experience With the COVID-19 Pandemic and Physical Distancing

Participants reported to feel, on average, moderately threatened by COVID-19 (Perceived Coronavirus Threat: *M* = 24.29; *SD* = 7.94)^1^ and not to have been heavily impacted by the pandemic in terms of financial security and mental health (Coronavirus Impacts scale: *M* = 26.95; *SD* = 10)^2^. Regarding their experience with COVID-19, only 1% of participants reported to have had first-hand experience with the virus, while 38% reported that somebody in their circle of acquaintances was diagnosed with COVID-19, often with direct health consequences (e.g., hospitalization, and in some cases death, see Table 3). Importantly, 91% of our sample affirmed to have been isolating for an average of 50 days at the time of the study (Table 4), and 60% reported to have had contact only with members of their household, which in one-third of the cases was just one person. Furthermore, 22% of the participants affirmed to have been isolating completely alone for more than 50 days at the time of the study.

**Table 4:** Participants' physical social distancing situation.

**Physical social distancing situation**		**Average days in isolation**	Average number of people in households^*^
Currently isolating	60%	M = 51.2, SD = 8.4	M = 1.7, SD = 1.5
Currently isolating but physical contacts with family/close friends	31%	M = 49.4, SD = 10.4	M = 2.3, SD = 2.1
Past isolation	1%	M = 35.4, SD = 37.3	M = 1.2, SD = 1.1
No current or past isolation	8%	—	—

* *Other than the participant*.

We also observed that longer time in isolation, as well as higher threat and impact of COVID-19, were associated with higher negative mood (days of isolation: *r* = 0.20, *p* = 0.024; COVID-19 threat: *r* = 0.43, *p* < 0.001; COVID-19 impact: *r* = 0.35, *p* < 0.001)^3^.

### Effect of Group Numerosity on the Appraisal of Social Scenes

**Figure 2:**
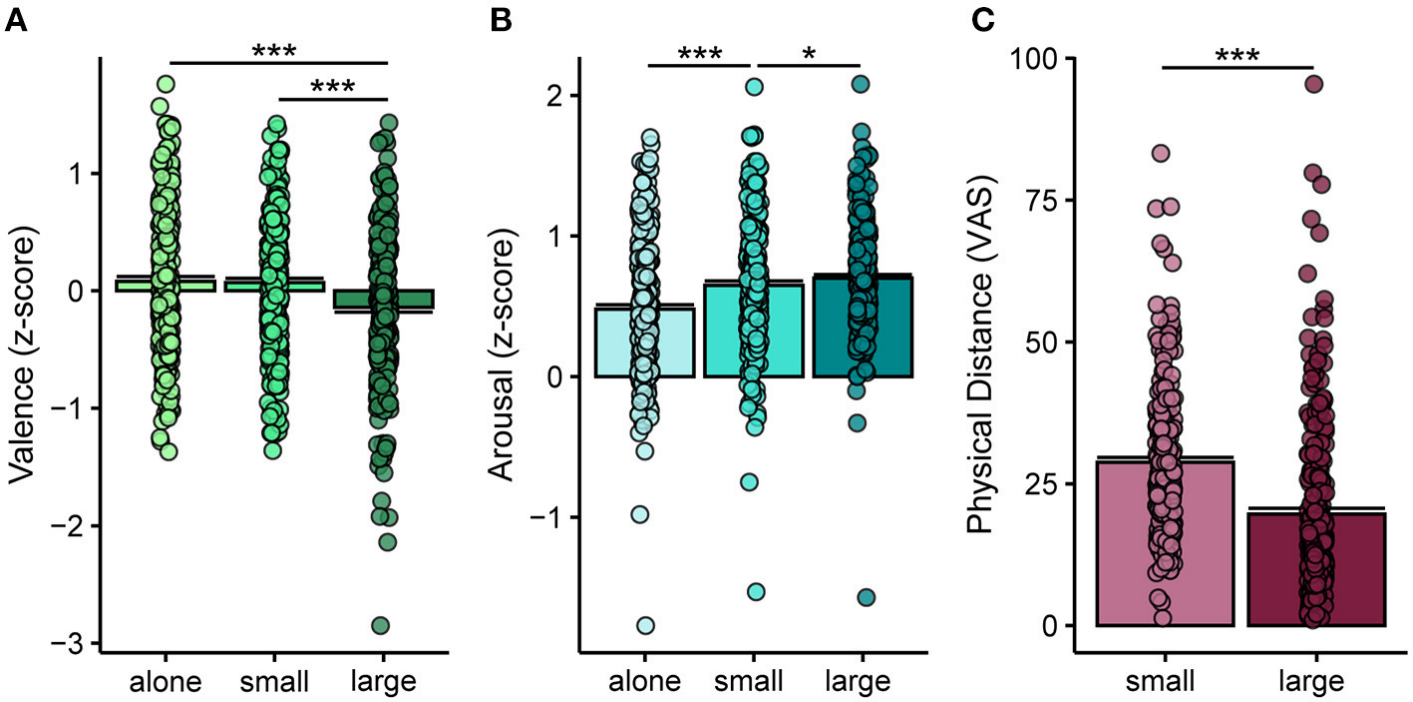
Mean ratings of **(A)** valence, **(B)** arousal, and **(C)** perceived physical distance. Ratings of valence and arousal were normalized by the average ratings provided by another group of participants collected before the COVID-19 pandemic (Kurdi et al., 2017). Perceived physical distance was only recorded in this study, and only in response to images depicting more than one person. Bars represent standard error of the mean; points represent individual means; asterisks indicate significant differences between conditions (^*^p < 0.05; ^***^p < 0.001).

To investigate whether the COVID-19 pandemic induced changes in the evaluation of social images, an LMM was fitted on the *z*-scores of valence (Figure 2A, Table 5). This revealed a significant main effect of Group Numerosity [*F*_(2, 235.98)_ = 26.95, *p* < 0.001]. As expected, participants rated the valence of images depicting individuals in large groups as significantly lower (more negative) compared to images of individuals alone and in small groups (both pairwise comparisons *p* < 0.001). Valence did not differ significantly between the alone and small group conditions (*p* = 0.9). To investigate whether the ratings of valence collected during the COVID-19 pandemic differed from the normative ones (OASIS dataset), we compared the *z*-scored ratings to zero using *t*-tests with Bonferroni correction. The analysis showed significantly reduced valence only for the images of large groups [*t*_(237)_ = −3.39, *p* = 0.003], while valence of images of individuals alone or in small groups did not differ significantly from zero (all *t* < 1.81, all *p* > 0.22).

**Table 5:** Mean (SD) of the normalized ratings (*z*-scores) of valence and arousal, and of the ratings of physical distance across the three categories of images (alone, small group and large group).

	**Alone**	**Small group**	**Large group**
Valence	0.08 (0.63)	0.07 (0.57)	−0.14 (0.65)
Arousal	0.48 (0.48)	0.65 (0.46)	0.7 (0.4)
Physical distance	—	28.80 (13.19)	19.67 (15.7)

Furthermore, the LMM fitted on the *z*-scores of arousal (Figure 2B, Table 5) revealed a significant main effect of Group Numerosity [*F*_(2, 236.75)_ = 47.39, *p* < 0.001]. Images of individuals in large groups were rated as more arousing than images of small groups (*p* = 0.035), which in turn were rated as more arousing than images of alone individuals (*p* < 0.001). Ratings of arousal for all the three conditions of Group Numerosity (alone, small group, and large group) were significantly higher during than before the pandemic, as indicated by *t*-tests of the *z*-scored ratings that were significantly greater than zero (all *t* > 15.66, all *p* < 0.001, Bonferroni corrected).

Lastly, the LMM fitted on the ratings of perceived physical distance (Figure 2C, Table 5) revealed a significant main effect of Group Numerosity [*F*_(1, 237.48)_ = 158.47, *p* < 0.001]. Individuals in large groups were perceived as physically closer compared to the individuals in small groups.

The pattern of the results for valence, arousal, and distance did not change after inclusion of the covariates age, nationality, country of residence, and participants' personal experience with COVID-19.

### Effects of Subjective Experience With COVID-19, Physical Distancing, and Loneliness on the Appraisal of Social Scenes

We had hypothesized that appraisal of social scenes, in particular images depicting large groups of individuals, would be modulated by the experienced risk associated with COVID-19, as well as by the degree of felt/experienced isolation. We therefore measured with self-reports loneliness (UCLA; Russell, 1996), social networks size (LSNS-6; Lubben et al., 2006), subjectively felt threat and impact of COVID-19 (Conway et al., 2020), changes in the form of social interactions (virtual and physical) and perceived relationship closeness (see Supplementary Material), as well as number of days in isolation.

First, we explored whether these collected measures were correlated and could be gathered in underlying factors. Therefore, an exploratory factor analysis was carried out which indicated the presence of four factors accounting for 69% of the total variance in the original measures. The rotated factor loadings showed that the number of days of isolation, changes in the perceived social closeness and perceived COVID-19 threat loaded on factor-1, which was named “Resilience” (loadings: 0.6, 0.67, 0.72). Positive values of this factor reflect more days of isolation and greater perceived COVID-19 threat, but also greater perceived closeness with significant others compared to the pre COVID-19 period, possibly indicating resilient response to the negative situation. Scores of loneliness and social network size loaded on factor-2 dubbed “Loneliness” (loadings: 0.85, −0.87). Positive values of this factor reflect smaller social network size and higher loneliness. Changes in physical and virtual social interactions loaded on factor-3, labeled “Changes in the form of social interaction” (loadings: −0.88, 0.76): positive values reflect an increase in virtual (but decrease in physical) interactions with others compared to the pre COVID-19 period, while negative values reflect an increase in physical (but decrease in virtual) interactions. Finally, perceived COVID-19 impact loaded on factor-4 (loading: 0.81). We termed this factor “COVID-19 impact”: positive values reflect greater negative psychological and financial impact of COVID-19.

Each of these four factors were then separately added as a fixed effect to the previous LMMs on valence, arousal, and perceived physical distance to investigate whether an eventual shift in those measures were associated to the subjective experience of the pandemic.

#### Resilience (Factor-1)

**Figure 3:**
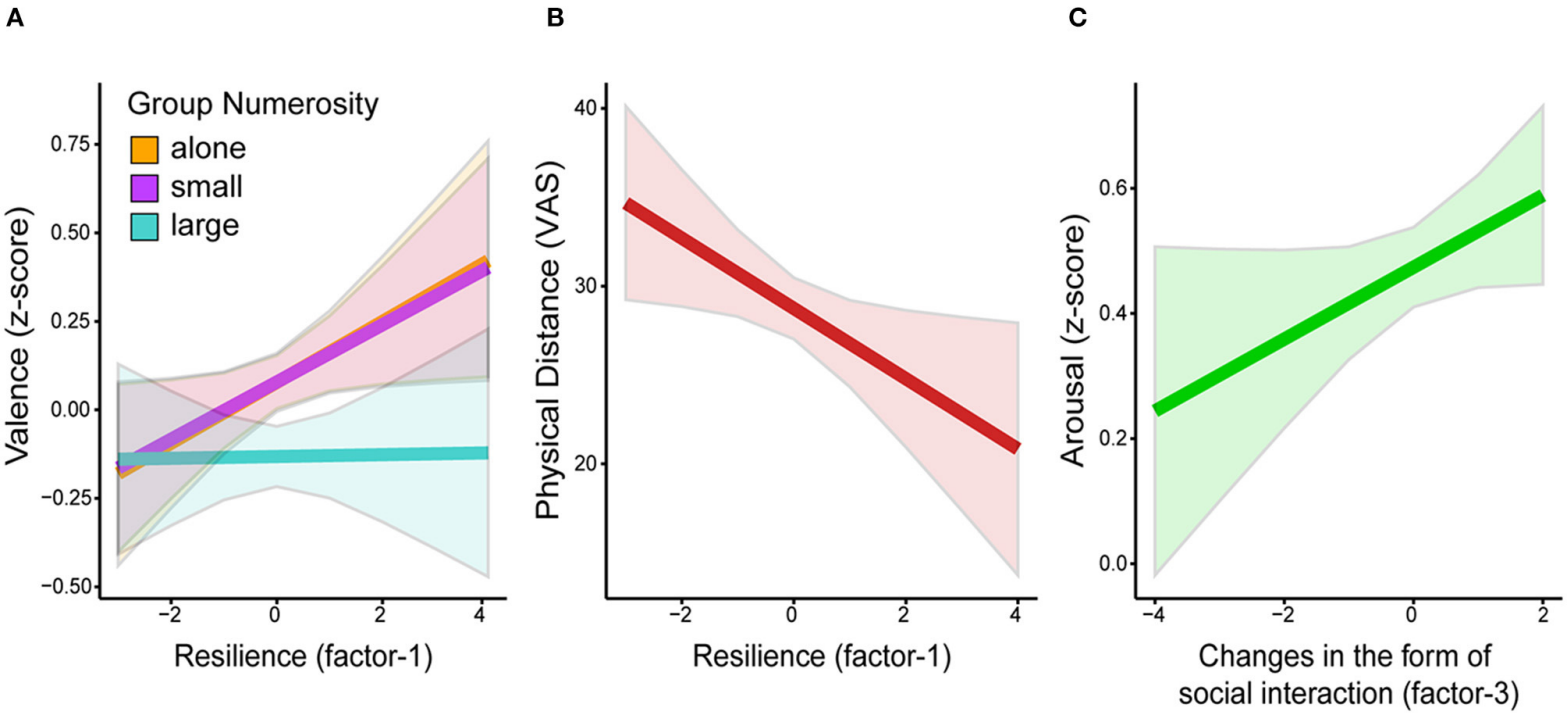
Predicted values (marginal means and 95% CIs) of **(A)** z-scores of valence by Group Numerosity and factor-1 (“Resilience”); **(B)** ratings of physical distance by factor-1; and **(C)** *z*-scores of arousal by factor-3 (“Changes in the form of social interaction”).

The LMM fitted on the *z*-scores of valence including the predictor Group Numerosity and the factor-1 “Resilience” revealed a significant main effect of Group Numerosity [*F*_(2, 213.08)_ = 24.23, *p* < 0.001] and a significant Group Numerosity by factor-1 interaction [*F*_(2, 213.25)_ = 3.45, *p* = 0.033]. As shown in Figure 3A, we observed a positive relationship between ratings of valence and this factor for images of individuals alone and in small groups, but not for images of individuals in large groups. Thus, the longer participants had been isolating, the more they perceived COVID-19 as a threat, and the closer they felt to their significant others, the more they liked pictures showing single individuals and small groups. Their liking of images of crowds, on the other hand, was not affected by this factor. The same LMM on the *z*-scores of arousal revealed only a significant main effect of Group Numerosity [*F*_(2, 213.45)_ = 46.72, *p* < 0.001]. The LMM on the ratings of perceived physical distance revealed significant main effects of Group Numerosity [*F*_(1, 214.41)_ = 169.91, *p* < 0.001] and of factor-1 [*F*_(1, 213.87)_ = 6.99, *p* = 0.009], indicating that participants scoring higher on this factor perceived the individuals depicted in the pictures as physically closer to one another (Figure 3B).

#### Loneliness (Factor-2)

The LMMs fitted on the *z*-scores of valence and arousal, and on the ratings of perceived physical distance, including the predictor Numerosity and the factor-2 “Loneliness,” revealed only a significant main effect of Group Numerosity [valence: *F*_(2, 212.97)_ = 23.53, *p* < 0.001; arousal: *F*_(2, 213.46)_ = 46.69, *p* < 0.001; physical distance: *F*_(1, 214.43)_ = 169.35, *p* < 0.001]^4^.

#### Changes in the Form of Social Interactions (Factor-3)

The LMM fitted on the *z*-scores of valence including the predictor Group Numerosity and the factor-3 revealed a significant main effect of Group Numerosity [*F*_(2, 213.03)_ = 23.5, *p* < 0.001]. The same LMM on the z-scores of arousal revealed a significant main effect of Group Numerosity [*F*_(2, 213.49)_ = 46.70, *p* < 0.001] and of factor-3 [*F*_(1, 214.14)_ = 5.22, *p* = 0.023]^5^, indicating that the pictures were generally perceived as more arousing by individuals who experienced a decrease in the number of physical social interactions during the pandemic as compared to the pre-pandemic period (Figure 3C). The LMM on the ratings of perceived physical distance showed only a main effect of Group Numerosity [*F*_(1, 214.41)_ = 169.19, *p* < 0.001].

#### COVID-19 Impact (Factor-4)

The LMM fitted on the *z*-scores of valence and arousal, and on the ratings of perceived physical distance, including the predictor Group Numerosity and the factor-4, revealed only a significant main effect of Group Numerosity [valence: *F*_(2, 213.99)_ = 23.49, *p* < 0.001; arousal: *F*_(2, 213.48)_ = 47.2, *p* < 0.001; physical distance: *F*_(1, 214.42)_ = 169.23, *p* < 0.001].

## Discussion

In the present study, we investigated the effects of the COVID-19 pandemic on the appraisal of social scenes. Between April and May 2020, images of individuals depicted alone, in small (up to four people), and large groups (more than seven people) were rated in terms of valence, arousal, and perceived physical distance. Valence and arousal were compared to pre-pandemic normative ratings (Kurdi et al., 2017). Further, we investigated if changes in these measures were associated with participants' personal experience of the COVID-19 pandemic.

Results indicate that during the pandemic images representing crowds and large gatherings were rated as less positive compared to the pre-pandemic period (Figure 2A). Moreover, participants experiencing overall greater physical isolation, stronger feelings of social closeness, and greater perceived threat of COVID-19 (factor-1) valued images depicting individuals alone and in small groups more positively (Figure 3A). Participants with higher scores on factor-1 also tended to judge the physical distance between individuals in small and large groups as smaller (Figure 3B). All three categories of images were rated as more arousing compared to the pre-pandemic normative data (Figure 2B). Further, higher arousal was associated with greater reduction of physical social interactions experienced during the pandemic (Figure 3C).

As humans, regular physical social contact with significant others, as well as with strangers, is part of everyday life, contributing to physical and psychological well-being (Field, 2010). The COVID-19 pandemic rapidly and drastically changed living habits and forced individuals to change their behavior, and the way they interact with others. Social activities involving gathering with other people, once considered harmless, such as going to a concert, suddenly had to be avoided to slow down the COVID-19 infection rate. We expected these changes to affect the thoughts and associations induced by the sight of unmasked crowds. Accordingly, we observed that during the pandemic the valence of images depicting large gatherings, which are associated with high risk for transmission of COVID-19, was significantly lower as compared to the pre-pandemic normative ratings of the very same pictures. Notably, the overall perceived valence of images of single individuals and small groups, which showed mainly intimate social interactions (e.g., partners, families), was not changed compared to pre-COVID-19 times. Further, all images were found to be more arousing than before the pandemic, with the highest increase for images of large groups. These findings point to a specific negative shift in participants' judgement of large gatherings during the COVID-19 pandemic, which may represent a possible mechanism to motivate avoidance of these potentially unsafe situations.

Further, we investigated whether these changes in the evaluation of social scenes were associated with the amount of physical distancing the participants had been exposed to, the perceived threat and financial/psychological impact of COVID-19, or changes in social behaviors and social connectedness. The results show that images depicting individuals alone and in small groups were rated more positively by participants who reported to be in isolation for a longer period of time, felt closer to their significant others compared to the pre-pandemic period, and felt more threatened by the COVID-19 pandemic (factor-1 “Resilience”). Valence ratings of images of crowds, on the other hand, were not modulated by this factor (Figure 3A). This finding suggests that participants who were more affected by the pandemic, both in terms of living (i.e., being forced to stay at home and avoid social contacts outside of the household for a large number of days) and psychological conditions (i.e., being more worried of a possible contagion for themselves and their relatives), had greater appreciation of images depicting situations considered safer in relation to the pandemic. Accordingly, across different cultures (Elmer et al., 2020; Killgore et al., 2020; Zhang and Ma, 2020) and pandemics (e.g., Lau et al., 2006), the caring for other family members and close friends increases, and the reciprocal support has been acknowledged to be an asset on which to rely for coping with the negative effects of a shared crisis. On the other hand, the reduced liking of crowded scenes during the pandemic (Figure 2A) was not affected by the participants' self-reported suffering from the pandemic (factor-1 “Resilience”; Figure 3A). The absence of modulation of this factor was not predicted. We speculate that this may be due to a floor effect, as the ratings of valence for images of individuals in large groups were already generally low, as shown in Figure 2A. As the COVID-19 pandemic represents a complex phenomenon impacting daily life on several aspects, it is also possible that other factors determined the lower valence expressed for this type of images, which we failed to capture with the collected self-report measures. Future studies measuring other aspects possibly altered by the pandemic (e.g., measures of health concerns or general compliance with governmental countermeasures) will be needed to assess this possibility.

The present results are also consistent within an evolutionary framework and the notion of behavior driven by archaic mechanisms of the immune system. Previous research has shown that the human immune system includes a behavioral component, evolved to discourage individuals from interacting with possible sources of infection by enhancing psychological mechanisms, such as disgust and fear (Schaller, 2006; Curtis, 2014; Troisi, 2020). Moreover, signals of possible disease result in social contact avoidance, and higher pathogen disgust sensitivity is related to higher anxiety and avoidance in response to stimuli associated with disease (Fan and Olatunji, 2013), as well as to lower social trust (Aarøe et al., 2016). Importantly, these mechanisms of pathogen avoidance seem to be less likely to affect social motivation toward significant others, such as relatives and close friends (Aarøe et al., 2016).

In the context of an imposed lockdown, it is worth to note that physical isolation and social isolation are not equivalent: being in physical isolation does not necessarily mean to feel lonely or socially distant to others (Abel and McQueen, 2020; Das Gupta and Wong, 2020). Indeed, in the identified factor-1 “Resilience”, the amount of days spent in physical isolation was positively correlated with the score in the scale “Changes in feelings of closeness”, indicating that individuals who lived longer in isolation also felt closer to their significant others compared to the pre-pandemic period. Previous studies had reported that lonely individuals perceive social scenes as less rewarding and more threatening (Cacioppo et al., 2000, 2009). We, however, did not find direct evidence for an effect of loneliness or social network size on the perceived valence of the images. We nevertheless observed that increased feelings of social closeness with significant others were comparable with the pre-pandemic period (included in the factor-1) were associated with increased valence of social scenes depicting individuals alone and in small groups, suggesting, in accordance with previous studies, an influence of social connectedness on the appraisal of social scenes.

Regarding the ratings of perceived physical distance, previous research has shown that it can be affected by a number of factors, such as perceived social distance (Won et al., 2018), social exclusion (Knowles et al., 2014; Pitts et al., 2014), motivation and desire (Balcetis and Dunning, 2010), as well as perceived threat (Cole et al., 2013). The pandemic has drastically changed our relationship with physical proximity, by associating it to the threat of infection. It forced us to keep physical distance during social interactions, and to keep others away from our personal space. We observed that longer time spent in physical isolation and higher perceived threat of the virus were associated with a smaller perceived distance between the individuals depicted in the images, indicating a biased perception of the environment and physical proximity as a consequence of the pandemic.

Some limitations of the current study need to be considered. First, although the collected sample was similar in terms of age and gender to the one included in the study from Kurdi et al. (2017), other factors, such as the different nationalities and countries of residence or the study design, might have influenced the results. Second, the sample collected was not homogenous in terms of country of origin. In spite of a similarity of lockdown and containment measures issued by the different governments at the time of data collection, differences still exist. Finally, the studied sample was mainly constituted of young adults and a full generalization to different age groups is therefore limited.

To conclude, the current findings provide evidence for changes in the appraisal of social scenes during the COVID-19 pandemic. The data reveal how regular social activities implying large gatherings once perceived as positive and harmless can rapidly assume a negative valence when external, highly impacting events, such as the 2020–2021 pandemic, occur. These changes may be part of a phylogenetically developed behavioral immune response, aimed at avoiding source of pathogen infection to maintain health and preserve survival. Future research should investigate the time course and long-term effects of this negative shift as the COVID-19 virus continues to represent a major threat for health across the world and containment measures, such as city lockdowns, are prolonged. Further, other pandemic-related factors which are likely to contribute to the appraisal of social scenes should be assessed. For instance, the use of face masks, which has already been found to contribute to changes in other social cognitive processes, like face perception (Freud et al., 2020), the ability to recognize emotions (Carbon, 2020) and the attribution of trust (Marini et al., 2021).

## Data Availability Statement

The datasets presented in this study can be found in online repositories. The names of the repository/repositories and accession number(s) can be found at: Open Science Framework (OSF), https://osf.io/mqau2/.

## Ethics Statement

The studies involving human participants were reviewed and approved by Ethics Review Board of the University of Amsterdam (2020-EXT-12259). The patients/participants provided their written informed consent to participate in this study.

## Author Contributions

CM, EC, RP, and SK contributed to conception and design of the study and data collection. CM performed the statistical analysis, under the guidance of EC, RP, and SK. CM and SK wrote the first draft of the manuscript. All authors contributed to manuscript revision, read, and approved the submitted version.

## Conflict of Interest

The authors declare that the research was conducted in the absence of any commercial or financial relationships that could be construed as a potential conflict of interest.

## Footnotes

^1^The maximum score of the Perceived Coronavirus Threat questionnaire is 42.

^2^The maximum score of the Coronavirus Impacts questionnaire is 63.

^3^All *p*-values were adjusted for multiple comparisons using Bonferroni correction.

^4^After including the covariates age, nationality, personal experience with COVID-19, and country of residence, we observed also a significant main effect of Loneliness on the *z*-scores of valence [*F*_(1, 201.13)_ = 4.32, *p* = 0.039] and on the ratings of physical distance [*F*_(1, 202.02)_ = 4.7, *p* = 0.031]. Ratings of valence decreased, while perceived physical distance increased, with higher scores of loneliness.

^5^After including the covariates age, nationality, personal experience with COVID-19 and country of residence this result was marginally significant (*p* = 0.053).
