# Supplementary material for: Large Gatherings? No, Thank You. Devaluation of Crowded Social Scenes During the COVID-19 Pandemic
Source: Front Psychol. 2021 May 31;12:689162. doi: 10.3389/fpsyg.2021.689162 (PMC8201791; doi:10.3389/fpsyg.2021.689162)
Supplement: Supplementary file 1 [file Data_Sheet_1.pdf]

Large gatherings? No, thank you. Devaluation of crowded social scenes during the COVID-19 pandemic

Claudia Massaccesi, Emilio Chiappini, Riccardo Paracampo, Sebastian Korb

- Supplementary Material –

Index

1. DETAILED DESCRIPTION OF THE SELF-REPORT ITEMS 2

1a. Changes in physical and virtual contacts with friends and relatives compared to the pre COVID-19 period 2

1b. Changes in feelings of closeness with friends and relatives compared to the pre COVID-19 period 2

2. DIFFERENCES WITH PRE COVID-19 STUDY 2

3. SUMMARY TABLES OF THE FITTED LMMS 3

4. TIMELINE OF COVID-19-RELATED GOVERNMENTAL MEASURES 7

4a. Austria 7

4b. Germany 8

4c. Italy 9

5. LIST OF IMAGES SELECTED FROM THE OASIS DATABASE (KURDI ET AL., 2017) 10

1. Detailed description of the self-report items

1a. Changes in physical and virtual contacts with friends and relatives compared to the pre COVID-19 period

The following questions explore how the relationship with your friends and family has changed after the implementation of social distancing measures in your country. Click and drag the slider to the desired position to answer the questions**.**

Compared to before social distancing measures were implemented by the government...

1. ...I have had physical (in person) contacts with my friends
2. ...I have had virtual contacts with my friends (e.g. via phone, videocall, etc.)
3. ...I have had physical (in person) contacts with my relatives
4. ...I have had virtual contacts with my relatives (e.g. via phone, videocall, etc.)

Ratings were expressed via a Visual Analog Scale (VAS) ranging from +1 (much less) to +101 (much more). Scores at two subscales and physical contact) were calculated averaging the ratings of the items 1-3 (physical contact) and 2-4 (virtual contact).

1b. Changes in feelings of closeness with friends and relatives compared to the pre COVID-19 period

The following questions explore how the relationship with your friends and family has changed after the implementation of social distancing measures in your country. Click and drag the slider to the desired position to answer the questions.

Compared to before social distancing measures were implemented by the government…

1. ...I feel my friends
2. ...I feel my relatives

Ratings were expressed via a VAS ranging from +1 (less close) to +101 (closer). A total score was calculated by averaging the ratings of the items 1 and 2.

2. Differences with pre COVID-19 study

Every participant in the pre COVID-19 study (Kurdi et al., 2017) had only rated a subset of the entire pool (i.e. 225/900). Notably, every stimulus included in the present study had been rated by a minimum of 100 individuals (valence and arousal rating range: 101-108 and 100-104). In contrast, our participants rated all the images, as we used a fully within-subjects design. In keeping, we decided to revise the analysis plan by firstly transforming our data in z-scores, and only then apply LMMs analyses.

3. Summary tables of the fitted LMMs

Effect of Numerosity on z-scores of Valence

	**z val**			
Predictors	*Estimates*	*CI*	*Statistic*	*p*
Intercept	0.00	-0.07 – 0.07	0.01	0.993
small group	-0.01	-0.03 – 0.02	-0.39	0.700
large group	-0.07	-0.09 – -0.05	-7.12	**<0.001**
**Random Effects**				
σ^2	1.05			
τ_{00} CASE	0.29			
τ_{11} CASE.condition1	0.02			
τ_{11} CASE.condition2	0.02			
ρ_{01}	-0.10			
	0.09			
ICC	0.24			
N CASE	238			
Observations	14228			
Marginal R^2 / Conditional R^2	0.007 / 0.244			

Effect of Numerosity on z-scores of Arousal

	**z aro**			
Predictors	*Estimates*	*CI*	*Statistic*	*p*
Intercept	0.61	0.56 – 0.66	23.59	**<0.001**
small group	0.09	0.07 – 0.11	8.59	**<0.001**
large group	0.05	0.03 – 0.06	6.69	**<0.001**
**Random Effects**				
σ^2	0.57			
τ_{00} CASE	0.15			
τ_{11} CASE.condition1	0.01			
τ_{11} CASE.condition2	0.01			
ρ_{01}	-0.00			
	-0.39			
ICC	0.23			
N CASE	238			
Observations	14224			
Marginal R^2 / Conditional R^2	0.012 / 0.239			

Effect of Numerosity on Distance ratings

	**distance**			
Predictors	*Estimates*	*CI*	*Statistic*	*p*
Intercept	24.23	22.53 – 25.93	27.94	**<0.001**
large group	-4.56	-5.27 – -3.85	-12.59	**<0.001**
**Random Effects**				
σ^2	376.55			
τ_{00} CASE	169.54			
τ_{11} CASE.condition1	21.76			
ρ_{01} CASE	0.30			
ICC	0.34			
N CASE	238			
Observations	9421			
Marginal R^2 / Conditional R^2	0.035 / 0.360			

Effect of Group Numerosity and Factor1 on z-scores of Valence

	z val			
Predictors	*Estimates*	*CI*	*Statistic*	*p*
Intercept	0.01	-0.06 – 0.08	0.22	0.822
small group	0.00	-0.03 – 0.03	0.03	0.975
large group	-0.07	-0.09 – -0.05	-6.65	**<0.001**
Factor1	0.06	-0.02 – 0.13	1.54	0.123
small group X Factor1	-0.00	-0.03 – 0.03	-0.18	0.860
large group X Factor1	-0.03	-0.05 – -0.01	-2.56	**0.010**
**Random Effects**				
σ^2	1.06			
τ_{00} CASE	0.27			
τ_{11} CASE.condition1	0.02			
τ_{11} CASE.condition2	0.02			
ρ_{01}	-0.10			
	0.08			
ICC	0.23			
N CASE	216			
Observations	12917			
Marginal R^2 / Conditional R^2	0.010 / 0.236			

Effect of Group Numerosity and Factor2 on z-scores of Valence

	z val			
Predictors	*Estimates*	*CI*	*Statistic*	*p*
Intercept	0.01	-0.06 – 0.08	0.22	0.823
small group	0.00	-0.03 – 0.03	0.03	0.975
large group	-0.07	-0.09 – -0.05	-6.56	**<0.001**
Factor2	-0.04	-0.11 – 0.03	-1.09	0.276
small group X Factor2	-0.01	-0.04 – 0.02	-0.74	0.461
large group X Factor2	-0.01	-0.03 – 0.01	-0.86	0.389
**Random Effects**				
σ^2	1.06			
τ_{00} CASE	0.27			
τ_{11} CASE.condition1	0.02			
τ_{11} CASE.condition2	0.02			
ρ_{01}	-0.11			
	0.05			
ICC	0.23			
N CASE	216			
Observations	12917			
Marginal R^2 / Conditional R^2	0.008 / 0.236			

Effect of Group Numerosity and Factor3 on z-scores of Valence

	z val			
Predictors	*Estimates*	*CI*	*Statistic*	*p*
Intercept	0.01	-0.06 – 0.08	0.22	0.823
small group	0.00	-0.03 – 0.03	0.03	0.974
large group	-0.07	-0.09 – -0.05	-6.56	**<0.001**
Factor3	0.02	-0.05 – 0.09	0.51	0.607
small group X Factor3	-0.03	-0.06 – -0.00	-2.00	**0.046**
large group X Factor3	-0.01	-0.03 – 0.01	-0.94	0.347
**Random Effects**				
σ^2	1.06			
τ_{00} CASE	0.27			
τ_{11} CASE.condition1	0.02			
τ_{11} CASE.condition2	0.02			
ρ_{01}	-0.09			
	0.06			
ICC	0.23			
N CASE	216			
Observations	12917			
Marginal R^2 / Conditional R^2	0.008 / 0.236			

Effect of Group Numerosity and Factor4 on z-scores of Valence

	z val			
Predictors	*Estimates*	*CI*	*Statistic*	*p*
Intercept	0.01	-0.06 – 0.08	0.22	0.823
small group	0.00	-0.03 – 0.03	0.03	0.975
large group	-0.07	-0.09 – -0.05	-6.55	**<0.001**
Factor4	-0.03	-0.11 – 0.04	-0.90	0.367
small group X Factor4	0.01	-0.02 – 0.04	0.63	0.527
large group X Factor4	-0.00	-0.02 – 0.02	-0.13	0.894
**Random Effects**				
σ^2	1.06			
τ_{00} CASE	0.27			
τ_{11} CASE.condition1	0.02			
τ_{11} CASE.condition2	0.02			
ρ_{01}	-0.10			
	0.05			
ICC	0.23			
N CASE	216			
Observations	12917			
Marginal R^2 / Conditional R^2	0.008 / 0.236			

Effect of Group Numerosity and Factor1 on z-scores of Arousal

	z aro			
Predictors	*Estimates*	*CI*	*Statistic*	*p*
Intercept	0.61	0.56 – 0.66	22.69	**<0.001**
small group	0.09	0.07 – 0.11	8.63	**<0.001**
large group	0.05	0.03 – 0.06	6.45	**<0.001**
Factor1	0.03	-0.02 – 0.09	1.25	0.213
small group X Factor1	0.00	-0.02 – 0.02	0.08	0.934
large group X Factor1	-0.01	-0.02 – 0.01	-0.97	0.330
**Random Effects**				
σ^2	0.57			
τ_{00} CASE	0.15			
τ_{11} CASE.condition1	0.01			
τ_{11} CASE.condition2	0.01			
ρ_{01}	-0.06			
	-0.41			
ICC	0.22			
N CASE	216			
Observations	12914			
Marginal R^2 / Conditional R^2	0.015 / 0.236			

Effect of Group Numerosity and Factor2 on z-scores of Arousal

	z aro			
Predictors	*Estimates*	*CI*	*Statistic*	*p*
Intercept	0.61	0.56 – 0.66	22.62	**<0.001**
small group	0.09	0.07 – 0.11	8.63	**<0.001**
large group	0.05	0.03 – 0.06	6.43	**<0.001**
Factor2	0.01	-0.04 – 0.06	0.44	0.662
small group X Factor2	-0.00	-0.02 – 0.02	-0.03	0.980
large group X Factor2	0.00	-0.01 – 0.02	0.30	0.765
**Random Effects**				
σ^2	0.57			
τ_{00} CASE	0.15			
τ_{11} CASE.condition1	0.01			
τ_{11} CASE.condition2	0.01			
ρ_{01}	-0.06			
	-0.42			
ICC	0.23			
N CASE	216			
Observations	12914			
Marginal R^2 / Conditional R^2	0.013 / 0.236			

Effect of Group Numerosity and Factor3 on z-scores of Arousal

	z aro			
Predictors	*Estimates*	*CI*	*Statistic*	*p*
Intercept	0.61	0.56 – 0.66	22.88	**<0.001**
small group	0.09	0.07 – 0.11	8.64	**<0.001**
large group	0.05	0.03 – 0.06	6.43	**<0.001**
Factor3	0.06	0.01 – 0.11	2.29	**0.022**
small group X Factor3	0.01	-0.01 – 0.03	0.62	0.538
large group X Factor3	-0.00	-0.02 – 0.01	-0.39	0.696
**Random Effects**				
σ^2	0.57			
τ_{00} CASE	0.14			
τ_{11} CASE.condition1	0.01			
τ_{11} CASE.condition2	0.01			
ρ_{01}	-0.07			
	-0.41			
ICC	0.22			
N CASE	216			
Observations	12914			
Marginal R^2 / Conditional R^2	0.018 / 0.236			

Effect of Group Numerosity and Factor4 on z-scores of Arousal

	z aro			
Predictors	*Estimates*	*CI*	*Statistic*	*p*
Intercept	0.61	0.56 – 0.66	22.61	**<0.001**
small group	0.09	0.07 – 0.11	8.69	**<0.001**
large group	0.05	0.03 – 0.06	6.44	**<0.001**
Factor4	-0.00	-0.06 – 0.05	-0.17	0.861
small group X Factor4	-0.02	-0.04 – 0.00	-1.66	0.098
large group X Factor4	-0.00	-0.02 – 0.01	-0.59	0.554
**Random Effects**				
σ^2	0.57			
τ_{00} CASE	0.15			
τ_{11} CASE.condition1	0.01			
τ_{11} CASE.condition2	0.01			
ρ_{01}	-0.07			
	-0.42			
ICC	0.23			
N CASE	216			
Observations	12914			
Marginal R^2 / Conditional R^2	0.013 / 0.236			

Effect of Group Numerosity and Factor1 on Distance ratings

	distance			
Predictors	*Estimates*	*CI*	*Statistic*	*p*
Intercept	23.99	22.26 – 25.72	27.14	**<0.001**
large group	-4.76	-5.47 – -4.04	-13.03	**<0.001**
Factor1	-2.34	-4.07 – -0.60	-2.64	**0.008**
large group X Factor1	-0.36	-1.07 – 0.35	-0.99	0.324
**Random Effects**				
σ^2	373.71			
τ_{00} CASE	159.29			
τ_{11} CASE.condition1	19.30			
ρ_{01} CASE	0.28			
ICC	0.32			
N CASE	216			
Observations	8550			
Marginal R^2 / Conditional R^2	0.049 / 0.356			

Effect of Group Numerosity and Factor2 on Distance ratings

	distance			
Predictors	*Estimates*	*CI*	*Statistic*	*p*
Intercept	23.99	22.24 – 25.74	26.89	**<0.001**
large group	-4.76	-5.47 – -4.04	-13.01	**<0.001**
Factor2	1.52	-0.23 – 3.27	1.71	0.088
large group X Factor2	-0.20	-0.92 – 0.51	-0.56	0.575
**Random Effects**				
σ^2	373.71			
τ_{00} CASE	162.46			
τ_{11} CASE.condition1	19.39			
ρ_{01} CASE	0.30			
ICC	0.33			
N CASE	216			
Observations	8550			
Marginal R^2 / Conditional R^2	0.043 / 0.356			

Effect of Group Numerosity and Factor3 on Distance ratings

	distance			
Predictors	*Estimates*	*CI*	*Statistic*	*p*
Intercept	23.99	22.23 – 25.75	26.71	**<0.001**
large group	-4.76	-5.47 – -4.04	-13.01	**<0.001**
Factor3	0.02	-1.74 – 1.79	0.03	0.978
large group X Factor3	-0.09	-0.81 – 0.63	-0.24	0.810
**Random Effects**				
σ^2	373.71			
τ_{00} CASE	164.80			
τ_{11} CASE.condition1	19.42			
ρ_{01} CASE	0.29			
ICC	0.33			
N CASE	216			
Observations	8550			
Marginal R^2 / Conditional R^2	0.039 / 0.356			

Effect of Group Numerosity and Factor4 on Distance ratings

	distance			
Predictors	*Estimates*	*CI*	*Statistic*	*p*
Intercept	23.99	22.23 – 25.75	26.75	**<0.001**
large group	-4.76	-5.47 – -4.04	-13.01	**<0.001**
Factor4	-0.74	-2.50 – 1.01	-0.83	0.406
large group X Factor4	-0.12	-0.83 – 0.60	-0.32	0.748
**Random Effects**				
σ^2	373.71			
τ_{00} CASE	164.24			
τ_{11} CASE.condition1	19.41			
ρ_{01} CASE	0.29			
ICC	0.33			
N CASE	216			
Observations	8550			
Marginal R^2 / Conditional R^2	0.040 / 0.356			

4. Timeline of COVID-19-related governmental measures

Summary of the major norms adopted by Austrian, German and Italian authorities from the outbreak to the 15th of May 2020 (i.e. end of data collection) to contrast the spreading of the COVID-19 and support financially the citizens. These measures and their announcement influenced the everyday life of our sample. They likely had a strong impact on how our sample perceived the current situation.

4a. Austria[1]

07-03	Suspension of flights and trains with northern Italy.
10-03	Home office is encouraged.
11-03	Closure of museums and cultural sites.
12-03	Hospital and hospice visits are prohibited.
13-03	Home office is highly encouraged. Restaurant must close at 15, shops closed. Paznauntal (Ischgl) e St. Anton/Alberg in Tyrol are quarantined.
15-03	Outdoor events > 500 people and indoor events > 100 people cancelled. Children >14 years ordered to stay at home. The government asked the public to **avoid social contact (physically at least 1 m distance)** and announced even further restrictions to be made soon. Ban for public gatherings > 5 people. Travel restrictions for people coming from Italy. Air connections to Spain, Switzerland and France are interrupted.
16-03	**Lockdown**, excluding: necessary professional activities, necessary purchases, assisting other people, activities outside, alone with same household. University classes suspended. Religious functions interrupted.
17-03	Children <14 years ordered to stay at home too. Restaurant closed. Borders closed to arrivals from Italy, China's Hubei Province, Iran, and South Korea.
19-03	Closure of public offices.
20-03	**Measures extension** until 13-04.
01-04	**PPE** mandatory in supermarkets (and controlled access).
06-04	**Announcement restriction loosening**:<br>From 14-04 small stores can reopen (if compliant with measures), mandatory PPE on public transport. From 01-05 large store can reopen. From 15-05 reopen restaurants, hotels (touristic), and schools. Public events not allowed until June.
14-04	Shops < 400 m^2 **re-opening**.<br>PPE mandatory in public transport.
21-04	Announcement:<br>Schools reopen from 04-05, all schools from 15-05; pubs and restaurants re-open from 15-05.
28-04	Announcement: from 01-05 no travel limitations within Austria borders, recommendation to keep at least 1 m social distance, **allowed gatherings with 10 people**, less limitations in stores, restaurants re-open from 15-05 (PPE for personnel).
13-05	(some) borders reopening from 15-06.
15-05	From 29-05 events <100 people allowed.<br>From 01-07 events <250 people people and cinemas can reopen.<br>From 01-08 events <500 people allowed, with derogation: up to 1000.

[1] http://coronavirus.quivienna.com

4b. Germany[2]

26-02	Regional closure of schools, public offices etc.
28-02	Quarantine for people showing symptoms or having been in contact with symptomatic people.
06-03	Health Minister recommends against unnecessary travel and suggests that people coming from risk areas stay at home.
08-03	Health Minister recommends to cancel events of more than 1000 attendees.
10-03	Ban on events with more than 1,000 participants.
13-03	14/16 States (Bundesländer) close schools.<br>Approved financial measures for people and companies struggling.
15-03	Certain national borders are being closed
17-03	**Limit to public movement** (interdiction of travelling in coaches, attending religious meetings, visiting playgrounds or engaging in tourism), closure of sports and leisure facilities, limitation to restaurants, non-essential shops are to be closed.
18-03	More **travel restrictions** abroad
21-03	Bavaria and Saarland **curfew**: allow work and supermarket shops, medics, outdoor sport, trip solitary or with housemates, visit partner or sick people not in facilities; Restaurants and shops closed.
23-03	**Forbidden gatherings** of >2 people and maintain a **distance of at least 1.5 m** in public except for families, partners or housemates. Restaurants and shops closed. Saxony curfew as Bavaria and Saarland.
02-04	Robert Koch institute recommends **PPE** for everybody, including people without symptoms.
15-04	**Announced reopenings**: 20-04 (if compliant with measures) of shops with a retail space of up to 800 m^2, bookshops, bike stores and car dealerships, allowed to; 04-05 of schools, hair salons; 31-08 large cultural events.<br>**Extension of social life restrictions** (see 23-03) until 03-05.<br>Recommendation (not mandatory) to wear **PPE** on public transport and while shopping.
20-04	**Shops start to reopen** (differences among Bundeslands in restrictions).
30-04	Opening of museums, monuments, botanical gardens and zoos, and religious services under strict social distancing conditions.
04-05	A maximum of 2 different households can meet in public. All shops are allowed to open, schools and kindergartens open in phases, people in care homes are allowed visits from one permanent contact person, outdoor sports without physical contact can resume. The decision on specific opening dates, including those for the restaurant sector, has been left to individual Bundesland.
10-05	Exceedance of the thresholds in 5 locations (Coesfeld, Rosenheim, Greiz, Sonneberg, Steinburg).
13-05	From 15-05 border controls with several neighboring countries would be loosened. From 15-06 free travel with Austria, France, and Switzerland.

[2] https://en.wikipedia.org/wiki/COVID-19_pandemic_in_Germany

4c. Italy[3]

30-01	Suspension of flights with China.
31-01	**Declaration of the state of emergency**.
21-02	Mandatory quarantine for who had contact with positive people or in areas at risk.
24-03	**3 red zones** in Lombardy and Veneto municipalities:<br>Forbidden the movement between municipalities, and organization of public cultural and sport events; schools, gyms, museums, shops closed; bars and restaurants open 6-18; home office strongly encouraged to businesses; **use of PPE** and keep **at least 1 m social distance**.
04-03	Schools are closed.
08-03	**Lockdown on some northern regions** affecting ~16M people until 03-04: Lombardy and parts of Emilia-Romagna, Piedmont, Veneto, Marche.
11-03	**Lockdown on the entire Italian soil** until 03-04. Also, no social gathering in public spaces, bars and restaurants closure.
16-03	Decree “cura Italia”: financial support to families, workers, enterprises.
20-03	**Forbidden movements** towards second houses, shutting down all non-necessary businesses and industries, access forbidden to parks and similar spaces.
23-03	Forbidden movements between municipalities, closed all non-essential activities.
01-04	**Measures extension** until 13-04
10-04	Announced:<br>**Lockdown extension** until 03-05, but from 14-04 small shops can re-open (bookshops, forestry, children's clothing).
26-04	Announced “phase-2” from 04-05
04-05	**Phase-2 (restrictions loosening)**:<br>Only motivated movements within region, more rigid if between regions. Reopened closed factories (no bars/restaurants, no barbers, no schools).<br>**Mandatory use of PPE** on public transport. Allowed access to parks. Funerals allowed if < 15 people. Mandatory quarantine and communication to health authorities as COVID-19 symptoms appear. Restaurants open for take away/delivery.
07-05	Announcement:<br>From 18-05 religious functions can resume.
13-05	Decree “rilancio”: financial support to families and business and taxes cut.

[3] http://www.governo.it/it/coronavirus-misure-del-governo

5. List of images selected from the OASIS database (Kurdi et al., 2017)

ALONE		SMALL GROUP		LARGE GROUP	
**Theme**	**Code**	**Theme**	**Code**	**Theme**	**Code**
beach_4	I62	Barbeque_1	I47	Band_1	I42
Biking_1	I72	Barbeque_2	I48	Camping_2	I105
Cliff_diver_2	I164	Birthday_2	I80	Child_labor_1	I153
Cold_6	I176	Camping_1	I104	Dancing_8	I206
Gazing_4	I392	Camping_4	I107	Exercise_2	I300
Gazing_6	I394	Children_1	I157	Exercise_3	I301
Guitar_1	I410	Cliff_diver_3	I165	Frisbee_1	I361
Hang_gliding_1	I422	Couple_2	I184	Parade_1	I608
Happy_pose_1	I428	Dancing_7	I205	performance_1	I617
Motocross_1	I514	Excited_face_3	I294	performance_2	I618
neutral_pose_2	I525	Excited_face_5	I296	Picnic_3	I622
Nude_man_1	I541	Hang_gliding_2	I423	Rafting_4	I656
Nude_man_3	I560	Nude_couple_7	I533	rollercoaster_1	I675
Parachuting_2	I605	Parachuting_3	I606	rollercoaster_2	I676
Parasailing_2	I610	Picnic_4	I623	Rugby_2	I686
Rock_climbing_2	I665	Rock_climbing_4	I667	School_1	I716
Rock_climbing_3	I666	Running_away_1	I687	School_2	I717
sailing_2	I705	sailing_1	I704	School_4	I719
Yoga_3	I897	Smiling_face_1	I766	School_8	I723
Yoga_4	I898	Soldiers_5	I786	soldiers_4	I785
